# Possible involvement of thiamine insufficiency in heart failure in the institutionalized elderly

**DOI:** 10.3164/jcbn.18-85

**Published:** 2019-02-06

**Authors:** Misora Ao, Kanae Yamamoto, Junko Ohta, Yasusei Abe, Naho Niki, Shino Inoue, Shinzo Tanaka, Akiko Kuwabara, Takashi Miyawaki, Kiyoshi Tanaka

**Affiliations:** 1 Department of Food and Nutrition, Kyoto Women’s University, 35 Kitahiyoshi-cho, Imakumano, Higashiyama, Kyoto 605-8501, Japan; 2 Faculty of Nutrition, Kobe Gakuin University, 518 Arise, Ikawadani-cho, Nishi-ku, Kobe, Hyogo 651-2180, Japan; 3 Nursing Care Home, Airanomori Ujigokasho, 19-1 Gokasho-tonouchi, Uji, Kyoto 611-0011, Japan; 4 Nursing Care Center, Care House Ajisai, 4-1-3 Kamo-cho-ekihigashi, Kizugawa, Kyoto 619-1154, Japan; 5 Nursing Care Center, Care House Yamabuki, 36-35 Ujisatojiri, Uji, Kyoto 611-0021, Japan; 6 Nursing Care Home, Villa Joyo, 1 Ichinobe-sasahara, Joyo, Kyoto 610-0114, Japan; 7 Graduate School of Comprehensive Rehabilitation, Osaka Prefecture University, 3-7-30 Habikino, Habikino, Osaka 583-8555, Japan

**Keywords:** heart failure, insufficiency, thiamine, brain natriuretic peptide, elderly

## Abstract

Heart failure is a major manifestation of thiamine deficiency; beriberi. Even thiamine insufficiency, milder than deficiency, may be associated with increased heart failure risk. In this cross-sectional study, the relationship between thiamine insufficiency and heart failure was investigated in the Japanese institutionalized elderly from April to November 2017. Fifty-five subjects in four care facilities were evaluated for their whole blood thiamine and plasma brain natriuretic peptide concentrations. Mean whole blood thiamine concentration was 88.7 ± 22.3 nmol/L in men and 92.0 ± 16.5 nmol/L in women, and significantly and negatively correlated with plasma brain natriuretic peptide concentrations (r = −0.378, *p* = 0.007). In the multiple regression analysis adjusted by age, sex, body mass index, and eGFR, whole blood thiamine concentration was a significant negative contributor (standardized coefficient β = −0.488, *p* = 0.001) to plasma brain natriuretic peptide. In the logistic regression analysis adjusted by the same variables, whole blood thiamine concentration significantly contributed to plasma brain natriuretic peptide concentration higher than over 40 pg/ml (OR: 0.898, 95%CI: 0.838–0.962). Whole blood thiamine concentration in subjects with diuretics was significantly lower than those without it (*p* = 0.023). Thiamine insufficiency was related to increased plasma brain natriuretic peptide concentration and may increase the risk of heart failure.

## Introduction

Thiamine deficiency causes beriberi, which consists of neurologic manifestation (dry beriberi) and cardiovascular manifestation (wet beriberi).^(1)^ Previously, beriberi was quite prevalent in Japan, and its severe form was lethal.^(2)^ Since thiamine is an essential cofactor in energy metabolism, it is quite natural that thiamine deficiency causes impaired myocardial function and heart failure.^(1)^ With the discovery of thiamine as well as the nutritional improvement of the society, the number of beriberi patients has dramatically decreased.^(3)^ Today, it seems to be generally considered that beriberi is a historical disorder and thiamine deficiency is no longer a serious health problem in Japan.

Vitamin deficiency causes classical deficiency diseases with typical phenotypic changes such as beriberi due to thiamine deficiency. In contrast, vitamin insufficiency, which is milder than vitamin deficiency, does not cause classical deficiency disease, but is associated with increased disease risk. For example, vitamin D deficiency causes rickets and vitamin D insufficiency is a serious risk factor for osteoporotic fracture.^(4)^

Compared to studies on the increased fracture risk by vitamin D insufficiency, reports have been scarce on the significance of thiamine insufficiency. Recently, however, papers from abroad have been published concerning the relationship between thiamine and diuretic therapy in patients with heart failure.^(5–9)^ Since thiamine does not have specific binding proteins, it is easily filtered in glomeruli and excreted in the urine, and the use of diuretics further leads to excessive loss of thiamine.^(10)^ Moreover, bodily storage of thiamine is quite limited.^(11)^ Then, a vicious circle is likely to occur, in which diuretics use causes thiamine deficiency and thiamine deficiency in turn impairs cardiac energy metabolism and further worsens heart failure.^(8)^

Japan has been becoming a super-aging society over the last few decades, and the number of patients with heart failure has been increasing rapidly.^(12)^ Then, identifying modifiable risk factors for elderly heart failure would be of great clinical and societal significance. Based on the above considerations, we have made a hypothesis that thiamine insufficiency is prevalent in the elderly and it is a risk factor for heart failure. As far as we have searched, there have been no reports investigating thiamine insufficiency in heart failure patients in Japan. Then, in this paper, we have studied the thiamine status and its possible association with heart failure in the institutionalized elderly, since they were considered to be at high risk for heart failure.

## Materials and Methods

### Study design and subjects

This is a cross-sectional study conducted from April 2017 to November 2017. The participants were fifty-five residents and users in four care facilities. Written consent to participate in this study was obtained from each subject after explanation of the objective and protocol of this study. The study protocol was approved by the Ethical Committee of Kyoto Women’s University (Ethics Approval number; 28-9), and the study was done complying with the Declaration of Helsinki.

Participants who were under oral thiamine administration were excluded. To exclude subjects with undeclared use of thiamine supplementation, we have also excluded subjects with their whole blood thiamine concentration above the third tertile + 1.5 × interquartile range;^(13)^ 146.3 nmol/L. A total of 51 (13 men, 38 women) subjects were included in this analysis.

### Biochemical measurements

Non-fasting blood was obtained. Serum, plasma or whole blood was stored at −70°C until measurement. Whole blood thiamine concentration was measured by liquid chromatography tandem mass spectrometry (LC/MS/MS), using UHPLC System (Shimadzu) with MS detector LCMS-8040. Inter-assay and intra-assay variation were 0.62 to 3.99% and 1.46 to 3.36%, respectively.^(14)^ Plasma brain natriuretic peptide (BNP) concentration was measured by chemiluminescent enzyme immunoassay (CLEIA). These measurements were done in Special Reference Laboratories (Tokyo, Japan). BNP is a cardiac hormone which is produced by ventricular myocardium in response to cardiomyocyte stretch. Its high levels reflect atrial/ventricular volume and pressure overload.^(15)^ Therefore, BNP is used as a biomarker for heart failure. Serum albumin concentration was measured by dye-binding (BCG) methods and serum total cholesterol concentration was measured by enzymatic (cholesterol oxidase) methods. Estimated glomerular filtration rate (eGFR; ml/min/1.73 m^2^) was calculated as 194 × creatinine^−1.094^ × age^−0.287^ for men and 194 × creatinine^−1.094^ × age^−0.287^ × 0.739 for women.^(16)^

### Statistical analyses

Statistical analyses were done using SPSS ver. 22 (IBM Japan, Tokyo, Japan). Pearson’s correlation was used to examine the relationship between whole blood thiamine concentration and plasma BNP concentration. The difference between two independent groups was analyzed by Student’s *t* test or Mann Whitney’s *U* test. The variables were analyzed using multiple regression analysis to assess the contribution of whole blood thiamine concentration for plasma BNP concentration. The association of whole blood thiamine concentration with the outcome; plasma BNP concentration over 40 pg/ml was examined by logistic regression analysis.^(17)^ For correlation analysis and multiple regression analysis, natural-log-transformed plasma BNP concentration was employed due to its skewed distribution. The significance level of the associations was set at *p*<0.05.

## Results

Fifty one individuals were included in this analysis (13 men, 38 women), and their characteristics are shown in Table 1. The mean age of participants was 84.2 years. Their BMI and serum concentrations of albumin were within the reference range, suggesting that this group was not generally malnourished. The mean eGFR was 57.3 ml/min/1.73 m^2^. Although it is slightly lower than the reference value of 60 ml/min/1.73 m^2^, it was considered to reflect the age-related decline of renal function.^(18,19)^ There was no significant gender difference in whole blood thiamine concentration. The number of subjects with diuretics use was 10 (3 men, 7 women), and it included both single use and multiple use. It consisted of loop diuretics (*n* = 5), angiotensin II receptor blocker with diuretics (*n* = 8), potassium holding diuretics (*n* = 3), and thiazide (*n* = 1).

### Correlation of whole blood thiamine and plasma BNP concentrations

Whole blood thiamine concentration has the significant negative correlation with plasma BNP concentration (r = –0.378, *p* = 0.007).

### Multiple regression analysis to assess the contribution of whole blood thiamine concentration for plasma BNP concentration

We have selected age, sex, body mass index (BMI) and eGFR to adjust this analysis. Whole blood thiamine concentration was a significant negative contributor (standardized coefficient β = −0.488, *p* = 0.001) (Table 2).

### Logistic regression analysis for suspected mild heart failure

We have examined whether whole blood thiamine concentration contributes to heart failure. Plasma BNP concentration higher than 40 pg/ml was used as an indicator for suspected mild heart failure. The adjustment was made with age, sex, BMI, and eGFR. There were 27 subjects with plasma BNP concentration higher than 40 pg/ml. Whole blood thiamine concentration significantly increased the risk of heart failure (OR: 0.898, 95%CI: 0.838–0.962) (Table 3).

### Comparison whole blood thiamine concentration between using diuretics and without it

When whole blood thiamine concentration in subjects with using diuretics was significantly lower than those without it (*p* = 0.023) (Table 4).

## Discussion

In this paper, we have studied whether thiamine insufficiency is associated with heart failure in the institutionalized elderly. These subjects were selected since they were considered at high risk for heart failure. Since our primary research question was whether even thiamine insufficiency, which is milder than deficiency, increases the risk of heart failure, we first wanted to make sure that they were not severely thiamine deficient. In this study, the mean whole blood thiamine concentration was 88.7 ± 22.3 nmol/L in men and 92.0 ± 16.5 nmol/L in women. Although established reference range for whole blood thiamine is not available, whole blood thiamine concentration in healthy Japanese college students was 104 ± 17 pmol/ml in men and 90 ± 23 pmol/ml in women.^(20)^ Elderly subjects have been reported to be prone to thiamine inadequacy.^(21)^ Considering that the average age of the current study subjects was 84.2, they were considered at least not to be thiamine deficient.

In our analysis, whole blood thiamine concentration contributed significantly and negatively to plasma BNP concentration in the multiple regression analysis, and it was a significant contributor to the plasma BNP concentration higher than 40 pg/ml in the logistic regression analysis. Of note, whole blood thiamine concentration in those with their plasma BNP concentration higher than 40 pg/ml was 84.3 ± 14.9 nmol/L, which is at least not severely decreased. Thus, thiamine insufficiency was considered to be associated with increased plasma BNP concentration and increased risk of heart failure.

Next, the possible involvement of diuretics use for the occurrence of thiamine insufficiency was considered. Whole blood thiamine concentration in subjects using diuretics was significantly lower than those without it. Thus, a vicious circle described below was considered likely to occur. Namely, thiamine deficiency/insufficiency leads to heart failure and patients with heart failure are usually prescribed diuretics. Since thiamine is easily filtered in glomeruli and excreted in the urine, when the volume of urine increases, loss of thiamine also increases and it causes deterioration of heart failure.^(10)^ Regarding the relationship between thiamine and diuretics in patients with heart failure, inconsistent results have been reported. According to a previous study, furosemide, which is a loop diuretic, causes increased urinary loss of thiamine.^(5)^ On the contrary, a cross-sectional study showed that there was no difference in the thiamine level between patients on loop diuretics and controls.^(6)^ Such discrepancy is probably due to the difference in the dosage and characteristics of subjects, and further studies are needed.

Although reports are limited to those from abroad, there have been intervention studies with thiamine in patients with heart failure. Previous reports including systematic review and metaanalysis have shown that thiamine supplementation significantly improved left ventricular ejection fraction (LVEF) which is a marker for the cardiac function.^(7,22–24)^ The prevalence of thiamine deficiency in congestive heart failure patients has been reported to range from 3% to 91%.^(8)^ Previous reports on the relationship between thiamine and heart failure have not been necessarily consistent. Many factors are likely to be responsible. In addition to the differences in measurement techniques, geographical differences in average daily dietary thiamine intake are also likely to be responsible.^(9)^

Thus, the possible association of thiamine nutritional status with heart failure must be studied in each country. In our literature search, we could not find papers from Japan on the possible involvement of thiamine insufficiency in the elderly heart failure even the observational ones. Then, we have carried out this cross-sectional study as a preliminary survey using plasma BNP concentration as a marker for heart failure.

Our study has several limitations. First, the fact that the study subjects were the institutionalized elderly has imposed some limitation. Nursing homes do not possess diagnostic equipment as available in hospitals, and we could not evaluate the subjects’ heart failure status using such sensitive indicators as LVEF, and we have employed plasma BNP concentration as the parameter of heart failure. Since most Japanese nursing homes are rather small-sized, number of subjects included in the current study remained only modest. Second, erythrocyte transketolase activity was not measured. Although this parameter has been reported to be sensitive markers for thiamine status,^(3)^ their measurement is quite difficult to perform. Finally, since this study was a cross-sectional study, causal relationship could not be concluded.

Nevertheless, our current data would be of clinical relevance. In recent decades, heart failure patients have increased rapidly in Japan, and it is expected to increase further in the future,^(25)^ which is due to the rapid increase of the elderly heart failure reflecting the super-aging society.^(12)^ Thus, identifying modifiable risk factor for elderly heart failure is an urgent need. As written above, however, there seem to have been no previous reports on the possible role of thiamine insufficiency for the elderly heart failure. Therefore, our present paper, although a preliminary one, would shed light on the modifiable risk for elderly heart failure. We are planning additional studies such as a cross-sectional study for non-institutionalized patients with heart failure, and intervention one for the patients with heart failure.

In conclusion, we have shown that blood thiamine concentration is a significant contributor to elevated plasma BNP concentration, and suggested the possibility that thiamine insufficiency is related to the heart failure in the elderly. Beriberi is generally thought to be almost overcome in Japan, and little attention seems to be paid on the role of thiamine in health promotion. Our results, however, suggest that even thiamine insufficiency can be a risk for elderly heart failure, and the role of vitamins in disease prevention must be re-emphasized.

## Author Contributions

MA was the principal investigator, and contributed to the conception and the design of the study with KT, MA, KY, JO, YA, NN, SI and ST were responsible for the evaluation the subjects’ general condition and nutritional status. MA, AK, TM and KT were responsible for statistical analyses. All authors have critically reviewed the final version of the manuscript and approved it.

## Figures and Tables

**Table 1:** Characteristics of the subjects

	Total (*n* = 51)	Men (*n* = 13)	Women (*n* = 38)	*p*
Age (year)	84.2 ± 6.6^†^	80.1 ± 9.1	85.6 ± 4.9	0.056
Body height (cm)	146.7 ± 8.9	155.0 ± 9.3	143.6 ± 6.6	<0.001
Body weight (kg)	44.3 ± 10.7	47.8 ± 9.9	43.0 ± 10.8	0.162
BMI (kg/m^2^)	20.5 ± 4.8	20.1 ± 4.0	20.7 ± 5.1	0.733
Serum albumin (g/dl)	4.1 ± 0.3	4.0 ± 0.3	4.1 ± 0.3	0.203
Total cholesterol (mg/dl)	189.1 ± 38.4	166.5 ± 34.6	196.8 ± 37.0	0.012
HDL cholesterol (mg/dl)	51.7 ± 12.8	45.9 ± 14.9	53.6 ± 11.6	0.061
LDL cholesterol (mg/dl)	103.7 ± 33.8	87.2 ± 35.5	108.9 ± 32.0	0.051
eGFR (ml/min/1.73 m^2^)	57.3 ± 15.7	44.9 ± 15.5	61.5 ± 13.5	0.001
Plasma BNP (pg/ml)	41.5^‡^ (17.4, 58.9)	51.7 (12.7, 100.8)	38.4 (17.6, 55.8)	0.381
Whole blood thiamine (nmol/L)	91.2 ± 18.0	88.7 ± 22.3	92.0 ± 16.5	0.573

^†^Data are expressed as mean ± SD and analyzed by Student’s *t* test except for BNP. ^‡^Data for BNP are expressed as median (Q1, Q3), and analyzed by Mann Whitney’s *U* test because of the skewed distribution.

**Table 2:** Multiple regression analysis to assess determinants for log-transformed plasma BNP concentration

Variables	Standardized coefficient β	95%CI	*p*
Age	0.348	0.001–0.041	0.042
Sex (ref: male)	−0.289	−0.577–0.083	0.138
BMI	−0.143	−0.035–0.011	0.312
eGFR	0.048	−0.007–0.010	0.783
Whole blood thiamine concentration	−0.488	−0.016–-0.005	0.001

Contributing factors for plasma BNP concentration were analyzed by multiple regression analysis, adjusted R^2^ = 0.276, *p* = 0.005.

**Table 3:** Logistic regression analysis for over 40 pg/ml of plasma BNP concentration

Independent variables	OR	95%CI	*p*
Age	1.160	0.969–1.389	0.106
Sex (ref: male)	0.085	0.004–1.752	0.110
BMI	0.908	0.765–1.079	0.274
eGFR	1.034	0.970–1.104	0.305
Whole blood thiamine concentration	0.898	0.838–0.962	0.002

Nagelkerke R^2^ = 0.549, *p*<0.001.

**Table 4:** Comparison whole blood thiamine concentration between using diuretics and without it

	Using diuretics (*n* = 10)	Without diuretics (*n* = 35)	*p*
Whole thiamine concentration	79.8 ± 15.1^†^	94.6 ± 18.1	0.023

^†^Data are expressed as mean ± SD and analyzed by Student’ *t* test.

## Abbreviations

BNP: brain natriuretic peptide
CLEIA: chemiluminescent enzyme immunoassay
eGFR: estimated glomerular filtration rate
LC/MS/MS: liquid chromatography tandem mass spectrometry
LVEF: left ventricular ejection fraction
